# Ablation of Y_1_ receptor impairs osteoclast bone-resorbing activity

**DOI:** 10.1038/srep33470

**Published:** 2016-09-20

**Authors:** Daniela M. Sousa, Francisco Conceição, Diana I. Silva, Luís Leitão, Estrela Neto, Cecília J. Alves, Inês S. Alencastre, Herbert Herzog, Paulo Aguiar, Meriem Lamghari

**Affiliations:** 1 Instituto de Investigação e Inovação em Saúde (i3S), Universidade do Porto, NanoBiomaterials for targeted therapies Group, Rua Alfredo Allen 208, 4200-135 Porto, Portugal; 2 Instituto de Engenharia Biomédica (INEB), Universidade do Porto, Porto, Portugal; 3 Instituto de Ciências Biomédicas Abel Salazar (ICBAS), Universidade do Porto, Porto, Portugal; 4 Faculdade de Medicina da Universidade do Porto (FMUP), Porto, Portugal; 5 Neuroscience Division, Garvan Institute of Medical Research, Darlinghurst, Sydney, NSW, Australia; 6 Centro de Matemática da Universidade do Porto, Departamento de Matemática da Faculdade de Ciências da Universidade do Porto (FCUP), Porto, Portugal

## Abstract

Y_1_ receptor (Y_1_R)-signalling pathway plays a pivotal role in the regulation of bone metabolism. The lack of Y_1_R-signalling stimulates bone mass accretion that has been mainly attributed to Y_1_R disruption from bone-forming cells. Still, the involvement of Y_1_R-signalling in the control of bone-resorbing cells remained to be explored. Therefore, in this study we assessed the role of Y_1_R deficiency in osteoclast formation and resorption activity. Here we demonstrate that Y_1_R germline deletion (Y_1_R^−/−^) led to increased formation of highly multinucleated (n > 8) osteoclasts and enhanced surface area, possibly due to monocyte chemoattractant protein-1 (MCP-1) overexpression regulated by RANKL-signalling. Interestingly, functional studies revealed that these giant Y_1_R^−/−^ multinucleated cells produce poorly demineralized eroded pits, which were associated to reduce expression of osteoclast matrix degradation markers, such as tartrate-resistant acid phosphatase-5b (TRAcP5b), matrix metalloproteinase-9 (MMP-9) and cathepsin-K (CTSK). Tridimensional (3D) morphologic analyses of resorption pits, using an in-house developed quantitative computational tool (BonePit), showed that Y_1_R^−/−^ resorption pits displayed a marked reduction in surface area, volume and depth. Together, these data demonstrates that the lack of Y_1_Rs stimulates the formation of larger multinucleated osteoclasts *in vitro* with reduced bone-resorbing activity, unveiling a novel therapeutic option for osteoclastic bone diseases based on Y_1_R-signalling ablation.

Osteoclasts are highly specialized multinucleated cells involved in bone resorption that derive from precursor cells of the hematopoietic lineage^1^. Bone-resorbing osteoclasts act in a concerted manner with bone-forming osteoblasts to accomplish bone remodelling, where old and damaged bone is resorbed followed by the formation of new bone^2^. The exacerbation of osteoclast-resorbing activity observed in cases of metabolic bone diseases (e.g. osteoporosis, Paget’s disease)^3^, metastatic bone disease^4^ and rheumatoid arthritis^5^, results in the formation of fragile bones, increased risk of fracture and disability, leading to serious pathological conditions and possibly to death. Hence, uncovering putative signalling pathways that control osteoclast activity is of vital importance and might unfold new promising treatment options.

Traditionally, bone remodelling is viewed as a complex process regulated by hormonal, autocrine/paracrine and mechanical signals. However, growing evidence has shown that signalling molecules supplied by skeletal sympathetic and sensorial nerve fibbers might also be directly involved in the control of bone turnover through neurotransmitter receptors expressed by bone cells^6^. Among the neurotransmitters expressed in bone microenvironment, described to modulate bone mass, emerges the neuropeptide Y (NPY) system through Y_1_R signalling pathway^7^,^8^,^9^.

Several findings supported a generalized and powerful peripheral action of Y_1_R signalling in the regulation of bone mass. Both germ line and osteoblast-specific Y_1_R deletion resulted in significantly greater cancellous and cortical bone volume in femoral, tibia, and vertebrae bones in mice^7^,^8^. Previous work from our group have also shown that the systemic blockade of Y_1_R signalling stimulates pronounced increments on bone mass^10^. These anabolic effects have been exclusively attributed to increased osteoblast activity, given that Y_1_Rs are expressed in differentiated osteoblasts^11^ and that the specific deletion of receptors from mature osteoblasts augmented osteoblast differentiation and activity^12^. Nonetheless, the involvement of Y_1_R signalling in bone turnover via osteoclast activity remains to be fully addressed. Y_1_R has been shown to be robustly expressed by immune cells (e.g. T-cells, monocytes and macrophages)^13^ and to play a pivotal role in macrophage function^14^. Since both osteoclasts and macrophages share a common progenitor lineage, these studies suggest a direct role for Y_1_Rs in osteoclast progenitors activity. Moreover, tartrate-resistant acid phosphatase (TRAP)-stained bone histological sections revealed an increase in osteoclast surface upon disruption of Y_1_R signalling in mice^7^,^10^.

In the light of these indications, we hypothesize that Y_1_R signalling pathway might also be involved in the regulation of osteoclast activity and function, thus acting in both arms of bone remodelling. Hence, the aim of this study was to investigate the functional role of Y_1_R signalling in osteoclastogenesis and bone matrix resorption, using bone marrow-derived osteoclasts retrieved from wild-type (WT) and Y_1_R germ line knockout (Y_1_R^−/−^) mice. Moreover, we have also developed a novel computational tool – BonePit – that allowed to perform a detailed morphologic analysis of the resorption pits produced by osteoclasts in a 3D space.

## Results

### Y_1_R gene expression is upregulated during osteoclastogenesis

**Figure 1:**
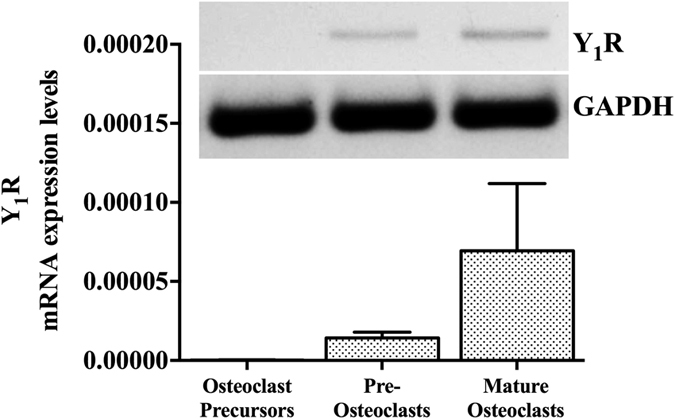
Y_1_R gene expression profile during osteoclastogenesis. The expression of Y_1_R is upregulated during osteoclast differentiation, as determined by qRT-PCR analysis. Values are normalized to GADPH levels (housekeeping gene). Data is expressed as mean ± SEM.

Previous studies have demonstrated that Y_1_^−/−^ mice retain a high-bone mass phenotype^7^. This feature has been associated with increased osteoblast activity and bone formation. Nevertheless little is known regarding the involvement of Y_1_R signalling in osteoclast function. Hence, we have first assessed the expression profile of Y_1_R during all stages of osteoclast differentiation^15^, using bone marrow retrieved from WT mice. The Y_1_R mRNA expression was demonstrated to be substantially upregulated throughout osteoclast differentiation (osteoclast fusion and maturation) by quantitative real-time PCR (qPCR) analysis (Fig. 1). As depicted in Fig. 1, Y_1_R gene expression increased considerably upon the differentiation of bone marrow-derived monocytes into small round mononucleated TRAP-positive cells (pre-osteoclasts) and the formation of multinucleated TRAP-positive cells (mature osteoclasts). These results provided the first indication that Y_1_Rs might play a regulatory function in osteoclast activity.

It is widely accepted that RANKL signalling pathway plays a role in osteoclast formation and differentiation^16^. Accordingly, the expression of RANKL and decoy receptor osteoprotegerin (OPG) was quantified by qRT-PCR analysis, using WT and Y_1_^−/−^ osteoblast cultures (Supplementary Information Fig. S1). Quantitative analysis revealed that RANKL gene expression is elevated in Y_1_R^−/−^ osteoblast cultures (p < 0.05), while no differences were detected for OPG when compared to WT cultures. Importantly, the RANKL/OPG ratio was significantly elevated in Y_1_R^−/−^ cultures (p < 0.001). These data confirmed our previous hypotheses involving the Y_1_R signalling pathway on osteoclastogenesis.

### Y_1_R signalling deficiency stimulates the formation of larger osteoclasts

**Figure 2:**
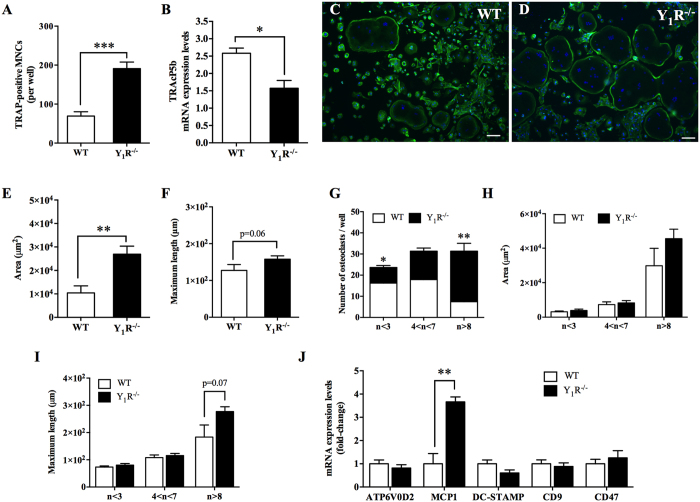
Y_1_R modulates osteoclast precursors fusion and osteoclast formation. The lack of Y_1_R signalling promoted an increase in the number of TRAP-positive multinucleated (MNCs) cells with more than 3 nuclei (**A**), while TRAcP5b mRNA expression levels (**B**) are decreased. The formation of podosome belts was observed in both WT **(C)** and Y_1_R^−/−^ (**D**) conditions. Quantitative analysis showed that Y_1_R^−/−^ osteoclasts have an elevated surface area (**E**) and a trend towards increased maximum length (**F**). Moreover, the number of osteoclasts with more than 8 nuclei is also elevated in Y_1_R^−/−^ cultures (**G**). Nevertheless, there were no differences between genotypes on osteoclast area (**H**) and in maximum length (**I**) per each category. The gene expression of key markers of migration and fusion revealed that MCP-1 is significantly upregulated, as determined by qRT-PCR analysis (**J**). Data is expressed as mean ± SEM. *p < 0.05, **p < 0.01, ***p < 0.001 different from WT. Scale bar = 100 μm.

To evaluate the specific role of Y_1_R in osteoclast differentiation, osteoclast precursors retrieved from WT and Y_1_R^−/−^ mice were stimulated in the presence of M-CSF and RANKL. As a first measure outcome, the number of TRAP-positive multinucleated cells (MNCs) with more than 3 nuclei was quantified. Y_1_R^−/−^ cultures showed a 2-fold increase in the number of TRAP-positive MNCs (with more than 3 nuclei) when compared to their WT counterparts (Fig. 2A; p < 0.001). Interestingly, although the number of TRAP-positive MNCs with more than 3 nuclei is increased in Y_1_R^−/−^ cultures, a trend towards decreased TRAcP5b activity (a marker of osteoclast activity) has been observed when compared to WT cultures (Fig. 2B; p = 0.1). Moreover, qRT-PCR analysis demonstrated that TRAcP5b expression levels were also reduced in Y_1_R^−/−^ osteoclasts (Fig. 2C; p < 0.05), suggesting Y_1_R^−/−^ osteoclast activity might be impaired.

The morphology of the WT and Y_1_R^−/−^ osteoclasts was then analysed by F-actin immunostaining (Fig. 2D,E). The formation of a characteristic podosome belt by the multinucleated cells was detected in both genotypes (WT *versus* Y_1_R^−/−^) in plastic plates. As shown in Fig. 2F,G, quantification analysis revealed a significant increase in the Y_1_R^−/−^ osteoclast surface area (p < 0.01) and a trend towards increased maximum length (p = 0.06), when compared to WT osteoclasts. These pieces of evidence indicate an increase in the size of osteoclasts in Y_1_R^−/−^ mice.

To characterize in more detailed the differences observed, multinucleated osteoclasts were grouped into three categories based on the number of nuclei per cell: a) n < 3; b) 4 < n < 7; and c) n > 8. As depicted in Fig. 2H, 53% of Y_1_R^−/−^ osteoclasts have more than 8 nuclei (n > 8) when compared to the 18% of WT osteoclasts (p < 0.01), and only 17% of Y_1_R^−/−^ osteoclasts have less than 3 nuclei (n < 3), when compared to 39% of WT osteoclasts (p < 0.05). Moreover, similar percentages were observed at the category 4 < n < 7 between the two groups. Nevertheless, no statistical differences between genotypes could be observed regarding the osteoclast area (Fig. 2I) and maximum length (p = 0.07; Fig. 2J) for each category, suggesting that the increase in Y_1_R^−/−^ osteoclast surface area and diameter is independent of the number of nuclei.

Taking into account that Y_1_R seems to stimulate the formation of larger multinucleated cells, we have then proposed to evaluate the expression of key markers of osteoclast precursors migration and fusion^17^ that might be upregulated in Y_1_R^−/−^ mice (Fig. 2K). Among the osteoclast fusion markers studied, quantitative analysis revealed that the expression of MCP-1 (also known as CCL2), known to be involved in osteoclast differentiation in a RANKL dependent manner^18^, was significantly elevated in Y_1_R^−/−^ osteoclasts (Fig. 2K; p < 0.01). Interestingly, no statistical differences were found in the gene expression of Atp6v0d2 (a component of the v-ATPase involved in osteoclast fusion that exerts its function through direct cell-cell contact), DC-STAMP (a RANKL-dependent transmembrane protein that is fundamental in osteoclast fusion), CD9 and CD47 (membrane proteins involved in cell fusion and cell recognition, respectively)^17^.

### Y_1_R deletion impairs matrix demineralization

**Figure 3:**
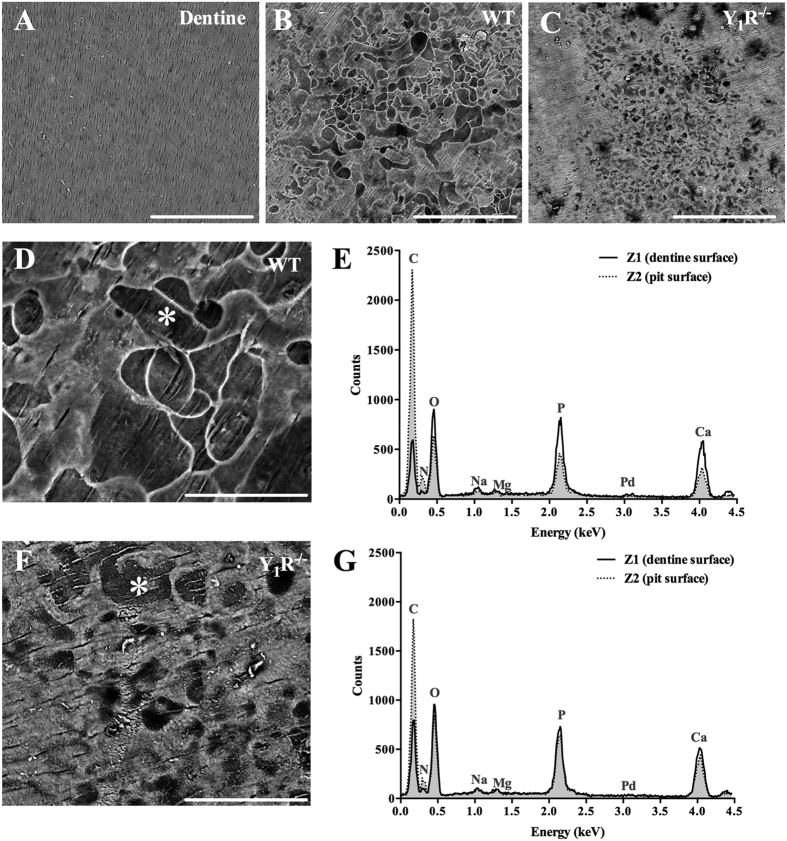
Y_1_R deficiency impairs the resorption of the mineralized surface. Osteoclast matrix resorptive activity was evaluated using dentine discs as substrates (**A**). Osteoclast precursors were seeded on top of dentine substrates. SEM analysis demonstrated that the lack of Y_1_R modulated the resorptive activity of osteoclasts resulting in a diminished eroded area, as depicted by representative micrographs of WT (**B**) and Y_1_R^−/−^ (**C**) dentine discs. EDS analysis of the dentine and pit (*) surfaces revealed a significant decrease in phosphate and calcium in WT eroded surfaces (**D,E**). These differences were not so prominent when comparing non-eroded and eroded Y_1_R^−/−^ surfaces (**F,G**). Scale bar (**A–C**) = 200 μm; Scale bar (**D,F**) = 50 μm.

To date, there are no published data reporting the specific effects of Y_1_R in osteoclast-resorbing capacity. In order to clarify this, osteoclast precursors were cultured on top of dentine substrate discs (untreated discs are displayed in Fig. 3A) in the presence of M-CSF and RANKL, to evaluate the resorbing activity of osteoclasts. These dentine discs are mainly constituted by a mineralized collagen matrix (collagen filaments covered by a hydroxyapatite layer) and are used in *in vitro* studies as bone resorption substrates^19^.

After culture, dentine substrates were scanned by electron microscopy (SEM). As shown by representative microphotographs in Fig. 3, Y_1_R^−/−^-cultured dentine substrates comprise a smaller region of resorptive area, and the resorption pits appear to be smaller and narrower (Fig. 3C,F), when compared to WT-cultured dentine substrates (Fig. 3B,D). Additionally, considering that darker regions correspond to exposed collagen, Y_1_R^−/−^ resorption pits are less deep than WT (as represented by brighter regions). To confirm these observations, the surface of the dentine discs was analysed by Energy dispersive X-ray spectroscopy (EDS) analysis (Fig. 3E,G), where the discrete energy of the backscattered electrons is used to roughly obtain the surface atomic composition up to few nanometers deep^20^. In both conditions, the dentine surface has been compared to the resorption pit surface (*), as illustrated in Fig. 3D–G. As expected, in WT dentine discs the phosphate and calcium peaks are elevated at the surface of the dentine, while the carbon peak is higher in the pit surface than in dentine surface (dashed line), most likely due to the exposure of collagen fibres each is mainly constituted by carbon (Fig. 3E). Interestingly, in Y_1_R^−/−^ dentine discs the differences observed in calcium and phosphate peaks in dentine and pit (dashed line) surfaces are not as steeper as for WT (Fig. 3G), suggesting an inferior exposure of collagen fibres and reduced matrix demineralization in dentin discs cultured with Y_1_R^−/−^ osteoclast precursors.

**Figure 4:**
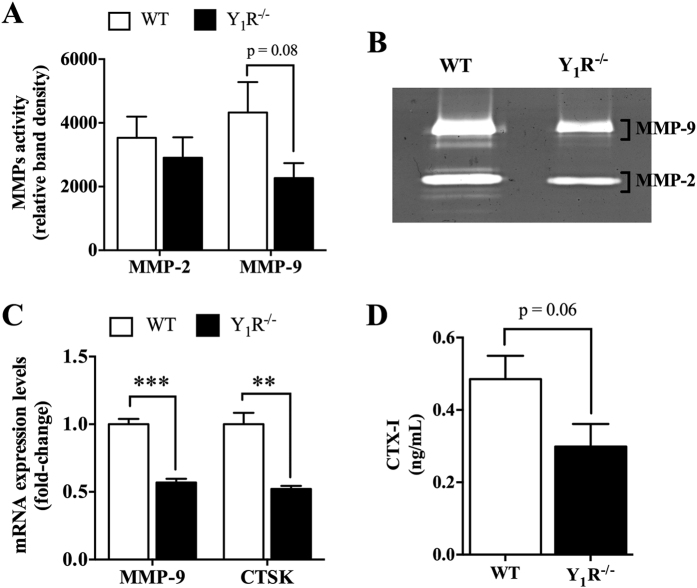
Y_1_R deficiency inhibits the expression of essential bone-resorbing factors. The proteolytic activity of MMP-9 in Y_1_R^−/−^ osteoclasts was slightly diminished, as determined by gelatin zymography (**A,B**). In agreement, the mRNA expression levels of MMP-9 and CSTK were also downregulated in Y_1_R^−/−^ cultures (**C**), which resulted in a trend towards decreased levels of released CTX-I (**D**). Data is expressed as mean ± SEM. **p < 0.01, ***p < 0.001 different from WT.

Subsequently, we have then explored the expression profile of key factors critical for the resorption of mineralized matrices, such as matrix metalloproteinases (MMPs) and CTSK, expressed by osteoclasts to degrade the organic components of bone matrix^21^,^22^. The quantitative analysis of gelatin zymography allowed us to demonstrate that there is a trend towards decreased MMP-9 activity in Y_1_R^−/−^ cultures when compared to WT (p = 0.08), while the proteolytic activity of MMP-2 was not different between groups (Fig. 4A,B). This was further confirmed by qRT-PCR analysis, where the MMP-9 mRNA expression levels were shown to be significantly downregulated in Y_1_R^−/−^ cultures (Fig. 4C; p < 0.001). In addition, the mRNA expression of CTSK was also diminished (Fig. 4C; p < 0.01). Furthermore, the quantification of degradation products from collagen I, such as C-terminal telopeptide (CTX-I) levels, revealed to be diminished in Y_1_R^−/−^ cultures when compared to WT (p = 0.06; Fig. 4D). Overall, the attenuation of collagen degradation correlates well with decreased matrix degradation, suggesting that bone matrix resorption might be impaired in Y_1_R^−/−^ cultures.

### Y_1_R deletion leads to the formation of small resorption pits

**Figure 5:**
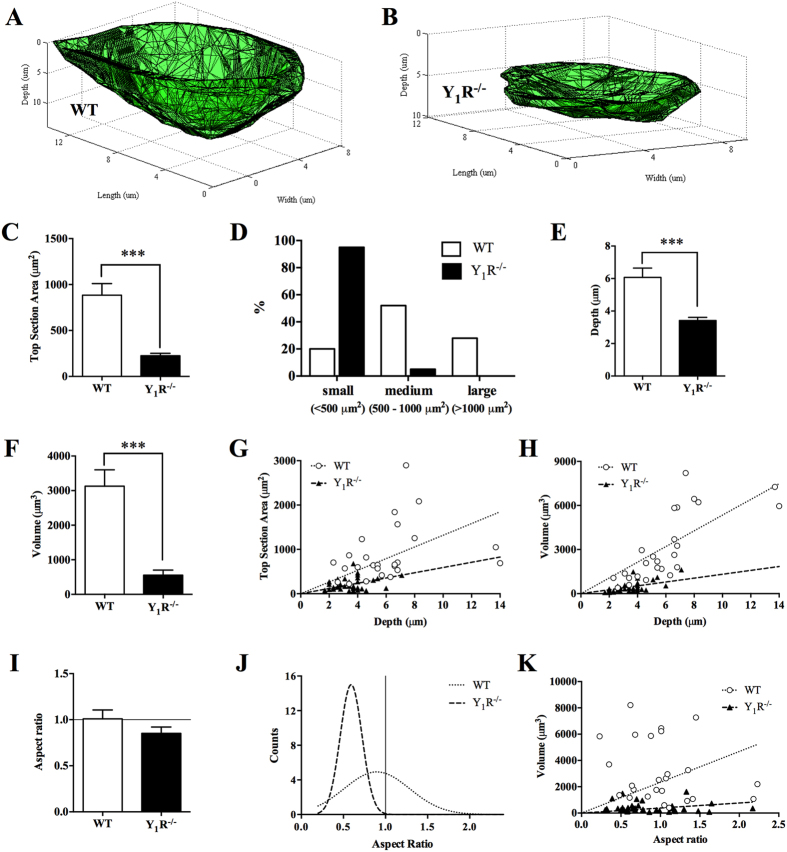
Y_1_R deletion impairs osteoclast resorption activity. The generation of 3D reconstructions of WT (**A**) and Y_1_R^−/−^ (**B**) resorption pits revealed striking morphological differences. The analysis of these 3D reconstructions demonstrated that Y_1_R^−/−^ resorption pits exhibit a decreased top section area (**C**), pit size (**D**), depth (**E**) and volume (**F**). Moreover, an increase on Y_1_R^−/−^ pit depth is not accompanied by augmented top section area (**G**) or volume (**H**). Although the mean aspect ratio of Y_1_R^−/−^ resorption pits was not different from WT (**I**), the frequency distribution of Y_1_R^−/−^ resorption pits revealed a shift towards less circular pits than WT (**J**), which did not alter with increases in pit volume (**K**). Data is expressed as mean ± SEM. ***p < 0.001 different from WT.

In order to better appreciate the differences observed in eroded surfaces between WT and Y_1_R^−/−^ cultures, we have developed a computational tool (BonePit algorithm) that enabled us to quantify in detail relevant pit morphologic features in a 3D space, such as pit top section area, volume, depth and aspect ratio. The 3D reconstruction of the resorption pits revealed striking morphological differences in the pits produced by WT (Fig. 5A) and Y_1_R^−/−^ osteoclasts (Fig. 5B). Quantitative analysis demonstrated that Y_1_R^−/−^ resorptive pits have a significantly smaller top section surface area (Fig. 5C; p < 0.001). To analyse these differences in detail, resorption pits were divided into three categories accordingly with their surface area: (a) small (<500 μm^2^); b) medium (500–1000 μm^2^) and (c) large (>1000 μm^2^). The percentage of resorption pits calculated per each category revealed that Y_1_R^−/−^ resorption pits are significantly small and medium sized, whereas WT pits are evenly distributed throughout all categories (Fig. 5D; p < 0.0001, Fisher’s exact test). In addition, based on a previously published scoring method by Caselli *et al.*^23^, the pit area index was estimated. Briefly, the number of pits of each category was multiplied by a factor: (a) small (x0.5), (b) medium (x1) and (c) large (x10). The sum of the three scores resulted in the pit area index. The Y_1_R^−/−^ samples presented a pit area index of 21, in comparison to a much elevated pit area index of 86 for WT resorption pits.

Furthermore, 3D analysis demonstrated that Y_1_R^−/−^ resorptive pits displayed a drastic reduction in volume (Fig. 5E) and in depth (Fig. 5F), when compared to WT resorption pits (p < 0.001). These marked results are consistent with the results obtained previously by SEM analysis (Fig. 3). Interestingly, by plotting the top section area *versus* pit depth, it is clear that the depth increase is not accompanied by an increase in the surface area in Y_1_R^−/−^ resorptive pits, resulting in a shift towards the bottom (Fig. 5G). A similar distribution can be observed in volume *versus* depth scatter (Fig. 5H), where a homogenous distribution is only visible for WT resorptive pits.

As an attempt to measure the circularity of the resorption pit, the maximum length of the pit was divided into the maximum perpendicular size, allowing conveying the “aspect ratio” of the resorption pits. An aspect ratio of 1 corresponds to an approximately circular section area. As shown in Fig. 5I,J, although there were no significant differences in resorption pits aspect ratio for both genotypes, it is clear that Y_1_R^−/−^ resorption pits are distributed far from the 1 value, in opposition to WT resorption pits. Moreover, increases in Y_1_R^−/−^ resorption pit volume are not associated with a circular aspect ratio (Fig. 5K). Together, these results suggest that osteoclast-resorbing capacity is impaired in Y_1_R^−/−^ mice.

To confirm that the inhibitory effects in osteoclastic resorption activity are Y_1_R-specific, osteoclast precursors were cultured in dentine substrate discs in the presence of increasing doses of a highly selective Y_1_R antagonist BIBP 3226 (0, 60, 1000 nM). Backscattered electron SEM analysis demonstrated that both doses of BIBP 3226 inhibit the formation of resorption pits, when compared to untreated osteoclasts (Supplementary Information Fig. S2). The resorption in 60 nM BIBP 3226-treated dentine discs appears to be more evenly distributed throughout the dentine surface but at a shallow depth, while in 1000 nM BIBP 3226-treated dentine discs suffered resorption within a smaller area, although resorption pits appear wider than at the lowest dose (Fig. S2). EDS spectra analysis comparing the dentine surface and pit surface confirmed that the peaks of phosphate and calcium remained reasonably unchanged at both treatment-conditions, when compared to untreated-dentine discs (Fig. S2).

Furthermore, resorption pit reconstructions revealed that BIBP 3226-treated dentine discs present a higher percentage of smaller resorption pits (p < 0.0001, Fisher’s exact test), regardless the concentration tested. Top section area, volume and depth (Fig. S2) of the resorption pits were also significantly diminished upon treatment. These results demonstrate that the Y_1_R antagonism recapitulates the effects of Y_1_R deletion in osteoclastic resorption activity, which suggests that the decrease in bone matrix resorption is Y_1_R-dependent.

## Discussion

It is known that Y_1_R^−/−^ mice exhibit a high-bone mass phenotype^7^, mainly due to elevated osteoblast activity^8^,^12^. Herein we demonstrate that the lack of Y_1_R promotes the formation of larger TRAP-positive multinucleated osteoclasts. Interestingly, these features were associated with diminished resorbing capacity, which was further confirmed by antagonizing Y_1_Rs using a highly selective antagonist BIBP 3226. Overall, these results suggest that the increase in bone mass observed in Y_1_R^−/−^ mice resulted from a deficiency on resorbing activity allied to augmented osteoblast activity.

Osteoclast differentiation and maturation have long been demonstrated to be regulated by RANKL/RANK/OPG signalling pathway^24^. RANKL stimulation leads to the formation and fusion of TRAP-positive MNCs^25^. NPY signalling pathway has been demonstrated to inhibit RANKL expression by MC3T3-E1 cells and osteoblast-derived bone marrow stromal cells^11^. Moreover, NPY has also been shown to inhibit RANKL isoprenaline-induced osteoclastogenesis, possibly through Y_1_R signaling^26^. In this study, we show that the lack of Y_1_R results in increased RANKL expression and RANKL/OPG ratio by osteoblasts. Therefore, we hypothesize that the presence of RANKL boosts the formation of larger multinucleated osteoclasts in Y_1_R^−/−^ cultures.

TRAcP5b is an acidic phosphatase highly expressed by the osteoclasts that is crucial for bone resorption. TRAcP5b is released into the resorptive-sealing zone and is partially responsible for bone demineralization process. The deletion of TRAP resulted in bone resorption impairment in mice^27^. Thus, TRAcP5b down-regulation can explain the observed reduction on bone resorbing activity in Y_1_R^−/−^ cultures. Furthermore, other study have also reported a correlation between osteoclast size and TRAP activity, where giant osteoclast cells showed decreased TRAP activity^28^, which is also consistent with our results.

The fusion of osteoclast precursors was also exacerbated in Y_1_R^−/−^ cultures, as seen by increased number of nuclei and surface area. Among the known osteoclast fusion markers, Y_1_R^−/−^ osteoclasts were shown to express high mRNA levels of MCP-1. Previous studies have demonstrated that the secretion of MCP-1 is significantly elevated in activated Y_1_R^−/−^ intraperitoneal macrophages *in vitro*, and it seems to be specific to Y_1_R signalling pathway^14^, which is in agreement with our data since both macrophages and osteoclasts derived from myeloid cells in the hematopoietic niche^1^. Moreover, MCP-1 is a chemotactic factor expressed by mature osteoclasts shown to promote osteoclast formation and fusion through cell-to-cell contact, under the control of RANKL signaling^18^,^29^,^30^. It is known that the exogenous treatment with MCP-1 in the presence of RANKL induces an increase in TRAP-positive MNCs formation^18^. Furthermore, the formation of multinucleated osteoclasts was significantly inhibited in MCP-1 knockout mice^29^. Thus, possibly the increase in TRAP-positive MNCs and osteoclast size in cells derived from Y_1_R^−/−^ mice might be due to an upregulation of MCP-1 expression through RANKL signalling. Overall, these data indicates that Y_1_R^−/−^ mice display increased formation of TRAP-MNCs possibly regulated by RANKL-signalling, and enhanced osteoclast fusion and surface area under the control of MCP-1. Nevertheless, future investigation is warranted to unravel the signalling pathways linking RANKL, MCP-1 and Y_1_R in osteoclast-mediated formation and fusion.

The RANKL expression is an important determinant of elevated bone resorption that is often associated with bone loss and osteoporosis^16^. In contrast, despite an increase in RANKL expression and an elevated number of TRAP-positive MNCs in Y_1_R^−/−^ cultures, Y_1_R^−/−^ mice still exhibit a high-bone mass phenotype. In point of fact, the pieces of evidence presented here suggest that the Y_1_R deficiency led to the formation of giant multinucleated osteoclasts with impaired resorbing capacity, possibly due to the lack of essential factors required for the normal function of bone-resorbing osteoclasts, such as CTSK and MMPs, as described previously^18^,^21^.

CTSK is a crucial enzyme in bone matrix resorption. The CTSK-deficiency has been associated with impaired osteoclastic bone matrix resorption^31^, resulting in the development of abnormal bone formation and osteopetrosis, which corroborate the observation that CTSK is downregulated in Y_1_R^−/−^ osteoclasts. Furthermore, the expression of MMP-9, a proteinase involved in collagen matrix degradation and bone resorption^21^, was also shown to be downregulated in Y_1_R^−/−^ cultures. Together, these observations confirm the formation of non-resorbing large multinucleated osteoclasts when Y_1_R signalling is disrupted.

Exploratory studies have implicated MMP-9 in the regulation of endochondral ossification using stabilized and non-stabilized fracture models^32^,^33^,^34^. MMP-9 was shown to be expressed throughout all phases of fracture repair, and its deficiency led to anomalous fracture repair^32^. Moreover, MMP-9^−/−^ mice displayed increased inflammatory populations at the fracture site and delayed hypertrophic cartilage turnover and matrix remodeling^33^. Furthermore, the broad inhibition of MMPs resulted in delayed endochondral ossification and fracture union in rats^35^. In the light of our findings, we speculate that the delay in hypertrophic cartilage removal and fracture union previously observed in Y_1_R^−/−^ mice^36^ might have occurred due to decreased MMP-9 expression and impaired osteoclast resorptive activity. Of note, independently of the facts presented, the endochondral growth and skeletal development appears to be uncompromised in Y_1_R^−/−^ mice^7^, which in turn suggests that albeit the resorption capacity has been affected in the absence of Y_1_R signalling, Y_1_R^−/−^ osteoclasts still sustain a residual resorptive activity.

Although osteoclast multinucleation has been commonly accepted to improve the resorption efficiency of mature osteoclasts^37^, it was previously observed that the volume resorbed per nucleus tend to decrease with the increase in nuclei number per osteoclast^38^. This was further explained by the fact that only a certain number of nuclei within multinucleated osteoclasts are transcriptionally active^39^, which support our observations that Y_1_R^−/−^ multinucleated osteoclasts exhibit decreased bone-resorbing activity regardless of the increased number of nuclei present.

Nevertheless, the intracellular signal transduction mechanisms triggered by Y_1_R deletion leading to the occurrence of these events remain to be uncovered. Y_1_R belongs to the family of G-protein coupled receptors (GPCRs). Knowledge gathered from previous studies demonstrated that Y_1_R is coupled to inhibitory heterotrimeric GTP-binding protein (Gi/Go)^9^. Its activation triggers a number of intracellular signal transduction cascades resulting in the: a) inhibition of adenylyl cyclase and cAMP; b) activation of phospholipase C and protein kinase C; c) elevation of cytosolic Ca^2+^; and d) activation of MAPK signalling pathway^9^. These transduction cascades are also known to be involved in osteoclastogenesis signalling cascades and in the regulation of MMPs and CTSK mRNA expression^40^,^41^, suggesting Y_1_R and osteoclastic signalling pathways (e.g. RANKL/RANK signalling) share common intracellular transduction pathways. Thus, we hypothesize that the disruption of Y_1_R intracellular signal cascade occurring in Y_1_R^−/−^ mice affected the pathways regulating the formation of osteoclasts and its resorptive activity. This hypothesis should be further explored in future research.

Recent studies have recognized that the geometry of resorptive cavities produced by osteoclasts (pits *versus* trenches) affect the structure, determining bone quality and its mechanical properties, such as bone stiffness and trabecular microarchitecture^42^,^43^. To date, the role of Y_1_R signalling deletion in bone quality and its impact on bone mechanical properties is still unknown. Future work should be undertaken to analyse the geometry of the resorption cavities produced by Y_1_R^−/−^ osteoclasts and evaluate the bone mechanical properties of Y_1_R^−/−^ mice.

In conclusion, our data demonstrates that Y_1_R plays an important role in osteoclast formation and activity, prominently in osteoclast resorptive capacity. The Y_1_R deficiency led to the formation of larger multinucleated cells with diminished resorbing capacity, resulting in a substantial reduction in resorption pits formation and eroded area. Taking into consideration that Y_1_R^−/−^ mice exhibit increased bone formation and in the light of the data presented we propose the use of Y_1_R antagonists as a clinically favourable therapeutic strategy for the treatment of osteoclast-related disorders, where bone resorption is chronically altered.

## Methods

### Animals

All research and animal care procedures were conducted following protocols approved by the Ethics Committee of the Portuguese Official Authority on Animal Welfare and Experimentation (DGAV). Mice were housed at 22 °C with a 12 h light/dark cycle with *ad libitum* access to water and food. All experiments were performed using 6–8 weeks-old male WT and Y_1_R^−/−^ mice. The generation of Y_1_R^−/−^ mice has been described in detail previously^7^,^44^. Y_1_R^−/−^ mice were backcrossed to a C57BL/6 background for at least five generations. Genotyping was performed using a PCR-based method to detect WT and knockout alleles^7^.

### Osteoclast cultures

Bone marrow cells were isolated from tibiae and femur (WT and Y_1_R^−/−^ mice) by flushing the bone marrow with α-MEM (Gibco, Thermo Fisher Scientific, USA) containing 10% (v/v) heat inactivated (30 min at 56 °C) foetal bovine serum (FBS, Gibco) and 1% (v/v) penicillin/streptomycin (P/S, Gibco)^11^. To generate primary osteoclast precursors, cell suspension was plated in the presence of 30 ng/mL M-CSF (PeproTech, USA) for three days. Adherent cells were then detached with 50 mM EDTA for 20 min at 37 °C and seeded in the presence of 30 ng/mL M-CSF and 100 ng/mL RANKL (PeproTech) at a density of 3 × 10^5^ cells/cm^2^. Cells were maintained at 37 °C in a humidified atmosphere of 5% CO_2_ for 4–5 days. To assess osteoclast resorptive capacity, osteoclast precursors were seeded on top of commercially available dentine substrate discs (5-mm diameter and 0.3-mm thick; OsteoSite Dentin Discs, Immuno Diagnostic Systems, UK) and allowed to generate resorption pits. At the end of the experiment, conditioned media were collected and stored at −80 °C before undergoing processing. Dentine substrates were prepared for electron microscopy and 3D quantitative analysis. All conditions were established in triplicates and repeated in 3 independent experiments.

### Quantitative CTX-I analysis

The levels of CTX-I in conditioned medium were quantified by enzymeimmunoassay (EIA) according to the manufacturer’s protocol (RatLaps^TM^ EIA CTX-I; Immunodiagnostic Systems, Denmark).

### TRAP staining

TRAP staining was performed using an Acid phosphatase, Leukocyte (TRAP) kit (Sigma-Aldrich) according to manufacturer’s instructions. Images were acquired using a stereomicroscope (SZX10, Olympus, Center Valley, PA, USA) coupled to a digital camera (DP21, Olympus). TRAP-positive multinucleated cells (MNCs) with more than 3 nuclei were counted.

### F-actin staining

Cells were incubated with Alexa Fluor^®^ 488 phalloidin (1:100; Molecular Probes) in 1% BSA for 20 min at RT to label the cytoskeletal filamentous actin (F-actin). Nuclei were stained with DAPI. Six representative images were obtained from each triplicate using an inverted fluorescence microscope (Carl Zeiss, Germany) coupled to a camera AxioCam HRc. The number of nuclei per cell was quantified. Osteoclast area and maximum length measurements were performed using the “measure outline” tool of the AxioVision SE64 Rel. 4.8 software (Carl Zeiss).

### SEM analysis

Dentine samples were fixed with 2.5% (v/v) glutaraldehyde in 0.1 M sodium cacodylate solution for 30 min and then washed with cacodylate buffer. Dentine discs were dehydrated in serially diluted ethanol solutions (50, 60, 70, 80, 90 and 99% v/v) for 10 min each dilution, and stored in absolute ethanol. Each sample was then critical point dried, sputtered-coated with gold and examined by SEM. Photomicrographs were obtained and the chemical composition of the dentine surfaces was evaluated by EDS analysis.

### Quantitative analysis of 3D resorption pits

**Figure 6:**
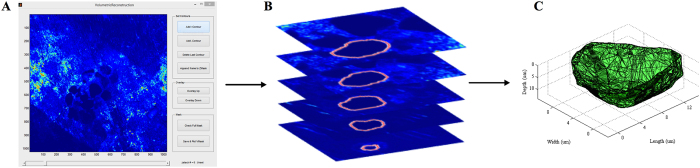
BonePit program – a computational tool for morphometric analysis of bone matrix resorption. Stacked images of calcein-stained dentine substrates are loaded into BonePit algorithm (**A**), developed in MATLAB. The program applies a Gaussian filter to reduce noise and changes the colour representation to identify more easily pit regions. The contours of a single pit were then manually drawn in each stack and compiled into a mask (**B**). Finally, every point in the mask is connected to the neighbouring points creating a 3D reconstruction mesh of the resorption pit (**C**).

Cultured dentine discs were washed with PBS and immersed in a 2 mM EDTA solution for 10 min at 37 °C to remove adherent cells. Dentine mineralized matrices were then stained with 10 μg/mL calcein AM (Molecular Probes, Life Technologies, USA) for 30 min, washed and allowed to air dry^19^. Images were obtained in a TCS SP2 spectral confocal and multiphoton system (Leica) at a resolution of 1024 × 1024 pixels with a 40x oil objective. Samples were exposed to a 488 nm radiation through a pinhole setting of 1 airy. Images of resorption pits acquired were averaged twice, and stacked images with a voxel size of 0.3662 × 0.3662 × 0.2849 μm were generated. The custom made program to perform the semi-automated 3D reconstruction of resorption pits was written using MATLAB 2013a (MathWorks, USA) software. As depicted in Fig. 6, stacked images were loaded into the program and the resorption pit outline was manually outlined in every stack. A mask containing every outline was then generated and used to reconstruct the resorption pit. From this 3D reconstruction it was possible to calculate the resorption pit volume, depth, top section area and aspect ratio. Volume was calculated by multiplying the number of pixels contained in the outlines by the voxel size. Depth was obtained by calculating the difference between the maximum layer index and the minimum layer index and multiplying by the step size (0.2849 μm). Top section area was calculated by multiplying the pixels in the first layer by the pixel area. Finally, the aspect ratio was defined as the ratio between the maximum length and the perpendicular maximum width of the pit. The complete set points associated with the resorption pit outlines were used to calculate a 3D mesh representing the pit surface (Fig. 6). Software details can be visualized and downloaded at *computools.i3s.up.pt/BonePit/*.

### Gelatin zymography

Conditioned medium was replaced by a serum-free medium 24 h prior to experiment endpoint. The media were loaded into gelatin–SDS polyacrylamide gels (Mini-Protean Tetra Cell system, Bio-Rad), as described previously^45^. Following electrophoresis, gels were stained with 0.1% w/v Coomassie Blue solution (Sigma-Aldrich). Clear bands of proteolytic degradation in contrast to a blue background of the gelatin substrate represent the activity of MMPs. Band densities were quantified by densitometric analysis using ImageJ software.

### qRT-PCR analysis

Total RNA was extracted using the Direct-zol™ RNA miniPrep according to the manufacturer’s protocol (Zymo Research, USA). RNA final concentration and purity (OD_260/280_) was determined using a NanoDrop 2000 instrument (NanoDrop Technologies, Thermo Fisher Scientific, Wilmington, Delaware, USA; NanoDrop 3.0.1 software). RNA was reverse transcribed into cDNA using the Superscript II reverse transcriptase kit (Invitrogen, Life Technology, Paisley, UK), according to the manufacturer’s protocol. qRT-PCR experiments were run using an iCycler iQ5 PCR thermal cycler (Bio-Rad Laboratories, Hercules, CA, USA) and analysed with the iCycler IQ^TM^ software (Bio-Rad; version 2.1). The specific primers used are described in Table 1. Target gene expression was quantified using the cycle threshold (Ct) values and relative mRNA expression levels were calculated as follows: 2^(Ct reference gene - Ct target gene). Mouse glyceraldehyde 3-phosphate dehydrogenase (GAPDH) was used as a reference gene. Both target and reference genes were amplified with efficiencies between 100 ± 5%.

### Statistical analysis

Data are expressed as mean ± standard error of the mean (SEM). Normal distribution of the data was assessed through Shapiro-Wilk test. Statistical differences between groups were compared using a two-way ANOVA and *post hoc* comparisons were performed with an independent samples t-test. When normal distribution was not verified, the non-parametric Kruskal-Wallis test followed by the Mann-Whitney U-test to assess statistical significance was performed. Statistical analysis was carried out with SPSS for Windows (version 20; SPSS Inc., Chicago, IL, USA) and statistically significance was accepted when p < 0.05.

## Additional Information

**How to cite this article**: Sousa, D. M. *et al.* Ablation of Y_1_ receptor impairs osteoclast bone-resorbing activity. *Sci. Rep.* **6**, 33470; doi: 10.1038/srep33470 (2016).

## Supplementary Material

Supplementary Information

## Figures and Tables

**Table 1:** Pairs of primers used in qRT-PCR analysis.

Gene	Forward Primer (5′- > 3′)	Reverse Primer (5′- > 3′)	Product Length (base pairs)
ATP6V0D2	TTCTTGAGTTTGAGGCCGAC	CAGCTTGAGCTAACAACCGC	144
CTSK	ATATGTGGGCCAGGATGAAAGTT	TCGTTCCCCACAGGAATCTCT	90
CD47	CGATGCCATGGTGGGAAACT	TCAGTGTTGAAGGCCGTGC	99
CD9	GCTGGGATTGTTCTTCGGGT	GCTTTGAGTGTTTCCCGCTG	171
DC-STAMP	AAGCGGAACTTAGACACAGGG	CAGCTAGGGCTTCGTGGAAA	101
GADPH	GCCTTCCGTGTTCCTACC	AGAGTGGGAGTTGCTGTTG	183
MCP-1	AGCCAACTCTCACTGAAGCC	GCGTTAACTGCATCTGGCTG	131
MMP-9	CGACTTTTGTGGTCTTCCCC	TAGCGGTACAAGTATGCCTCTG	83
OPG	GTGGAATAGATGTCACCCTGTGT	TTTGGTCCCAGGCAAACTGT	110
RANKL	CCCATCGGGTTCCCATAAAGT	AGCAAATGTTGGCGTACAGG	114
TRAcP5b	CGACCATTGTTAGCCACATACG	TCGTCCTGAAGATACTGCAGGTT	77
Y_1_R	CTCGCTGGTTCTCATCGCTGTGGAACGG	GCGAATGTATATCTTGAAGTAG	325

All primers used were located on two different exons to ensure that only properly spliced mRNA and not genomic DNA contaminants was amplified.
