# Supplementary material for: Ablation of Y1 receptor impairs osteoclast bone-resorbing activity
Source: Sci Rep. 2016 Sep 20;6:33470. doi: 10.1038/srep33470 (PMC5028844; doi:10.1038/srep33470)
Supplement: Supplementary Information [file srep33470-s1.pdf]

Supplementary Information

Ablation of Y_1 receptor impairs osteoclast bone-resorbing activity

Daniela M. Sousa, Francisco Conceição, Diana I. Silva, Luís Leitão, Estrela Neto, Cecília J. Alves, Inês S. Alencastre, Herbert Herzog, Paulo Aguiar and Meriem Lamghari

Materials & Methods

Osteoblast cultures

Primary calvaria osteoblasts were harvested from newborn mouse pups with up to 6 days, after consecutive digestions with 0.1% collagenase (Sigma-Aldrich, USA) and 0.2% dispase II (Roche Technologies, France) at 37ºC. After expansion, primary calvaria osteoblasts were plated at a density of 2.5 x 10^4 cells/cm^2 in the presence of 50 µg/mL ascorbic acid 2-phosphate (Sigma-Aldrich), 10 mM β-glycerophosphate, 10 nM dexamethasone (Sigma-Aldrich), 1 µM prostaglandin E2 (PGE2; Sigma-Aldrich) and 10 nM 1,25-dihydroxyvitamin D3 (calcitriol; Sigma-Aldrich). The medium was refreshed every 3-4 days, for a total period of 14 days. At the end of experiment, the gene expression of RANKL and OPG was evaluted by RT-PCR analysis.

Osteoclast cultures

WT osteoclast precursors were culture on top of dentine substrates and treated with increasing concentrations (0, 60 and 1000 nM) of a highly selective Y_1 receptor antagonist BIBP 3226 (Tocris Bioscience). Dentine were prepared for SEM and 3D quantitative analysis.

Figure S1

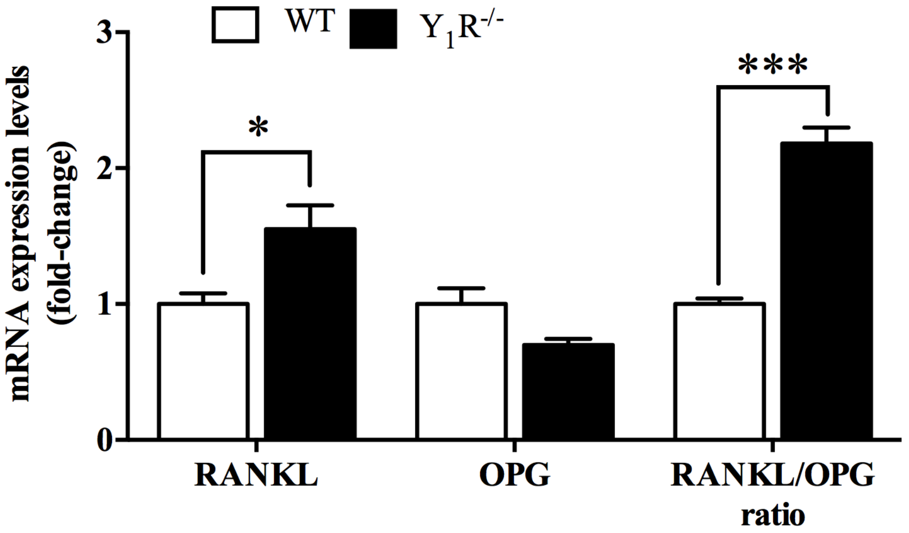

Figure S1 - Y_1R deficiency stimulates the expression of RANKL by osteoblasts. $Y_1R^{-/-}$ osteoblasts were shown to express high levels of RANKL mRNA levels with increased RANKL/OPG ratio, suggesting a role for Y_1R signalling in osteoclastogenesis. Values are normalized to GADPH levels (housekeeping gene). Data is expressed as mean ± SEM. *p<0.05, ***p<0.001 different from WT.

Figure S2

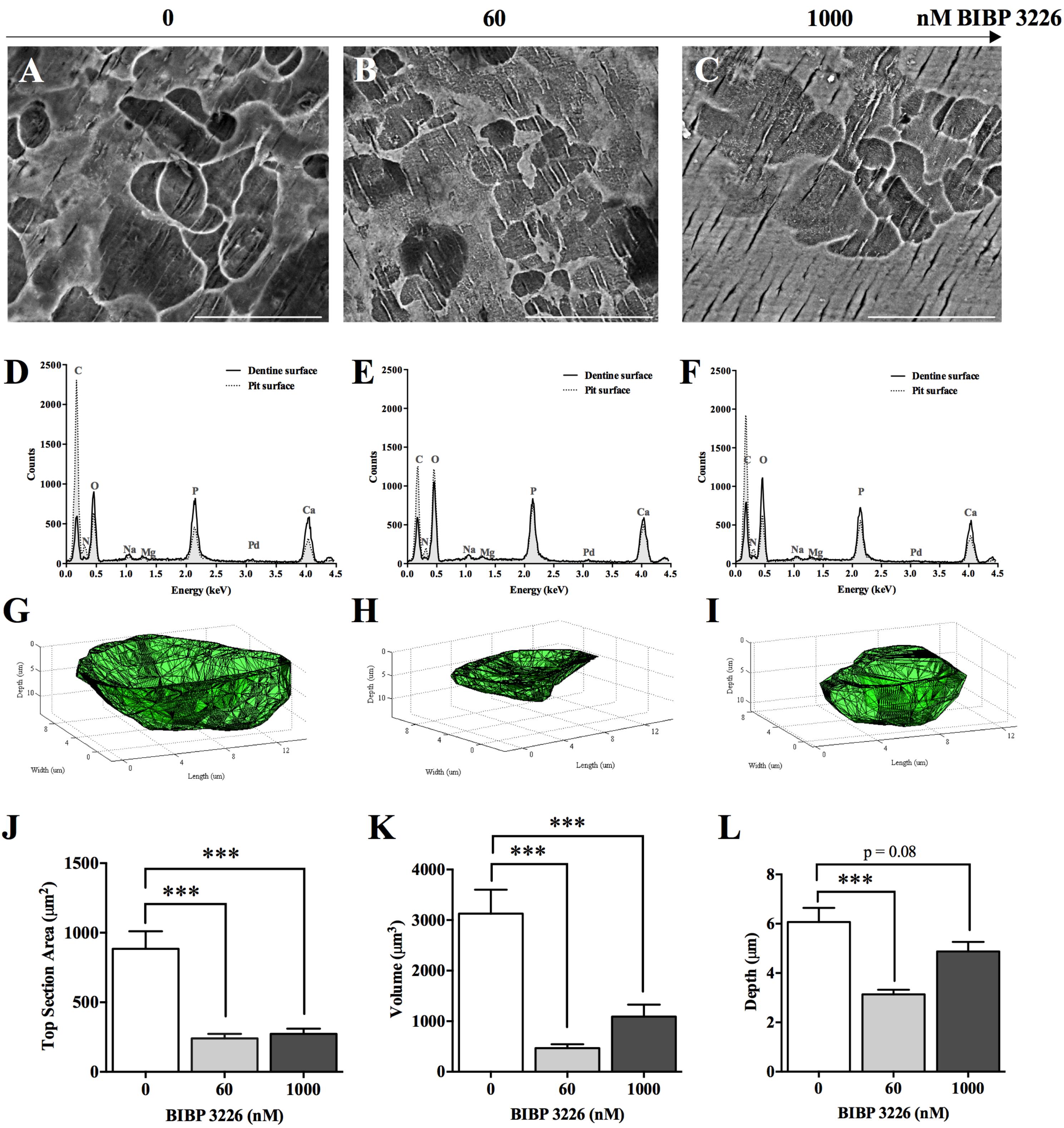

Figure S2. Y_1R antagonism decreases demineralization rate. BIBP 3226-treatment resulted in a decreased eroded area of dentine substrates, as depicted in representative SEM images (A-C). EDS spectrum analysis revealed that when compared to untreated substrates (D) calcium and phosphate peaks in eroded surfaces remained unaltered with 60 nM or 1000 nM BIBP 3226-treatment (E-F). The morphological analysis of 3D reconstructions (G-I) demonstrated that BIBP 3226-treatment induces a decrease in pit area (J), volume (K) and depth (L). Data is expressed as mean ± SEM. ***$p<0.001$ different from untreated cells. Scale bar = 50 µm.
